# HLA alleles and dengue susceptibility across populations in the era of climate change: a comprehensive review

**DOI:** 10.3389/fimmu.2025.1473475

**Published:** 2025-04-15

**Authors:** Amit Gourav Ghosh, Hie Lim Kim, Seik-Soon Khor

**Affiliations:** ^1^ Singapore Centre for Environmental Life Sciences Engineering, Nanyang Technological University, Singapore, Singapore; ^2^ GenomeAsia 100K Consortium, Singapore, Singapore; ^3^ Asian School of the Environment, Nanyang Technological University, Singapore, Singapore

**Keywords:** dengue, HLA, dengue fever (DF), dengue haemorrhagic fever, serotype, climate change

## Abstract

Dengue, a viral infection transmitted by *Aedes* mosquitoes, is an emerging global health threat exacerbated by climate change. Rising temperatures and altered precipitation patterns create favourable conditions for vector proliferation and extended transmission periods, increasing the risk of dengue in endemic regions and facilitating its spread to non-endemic areas. Understanding the interplay between critical genetic factors and dengue susceptibility is crucial for developing effective public health strategies. The *Human Leukocyte Antigen (HLA)* genes encode proteins essential for an effective immune response against pathogens, and their genetic variations influence susceptibility to severe dengue. In this study, we conducted a comprehensive meta-analysis of *HLA* alleles associated with dengue infection and dengue severity. We analysed 19 case-control studies on dengue infections in populations worldwide to infer *HLA* associations with various pathological forms of dengue and to examine differences across different populations. Our findings indicate that HLA-A*02 increases susceptibility to dengue fever (DF), while HLA-A*03 increases the risk of Dengue Haemorrhagic Fever (DHF), with these increased susceptibilities primarily observed in Southeast Asian populations. Additionally, HLA-A*24 is associated with DHF and all symptomatic dengue infections (DEN), contributing to dengue risk in both Southeast Asia and the Caribbean. Conversely, HLA-A*33 and HLA-B*44 show a protective effect against DHF but show significant regional heterogeneity, highlighting divergent, population-specific susceptibility profiles. This study underscores the importance of population-specific genetic risk assessments for dengue infection and emphasizes the need for targeted medical interventions and improved predictive models to mitigate dengue’s impact, especially as climate change accelerates disease spread.

## 1 Introduction

### 1.1 Dengue – dengue viruses and pathogenesis

Dengue is a viral infection emerging as a significant threat to global health, caused by the dengue virus (DENV). DENV is primarily transmitted through a human-to-mosquito-to-human cycle by specific mosquito vectors known as *Aedes aegypti* and *Aedes albopictus*. In the year 2023, the number of reported dengue cases reached a historical high of 6.5 million (1); as of 30 April 2024, the total number of cases had reached a staggering 7.6 million (2) for the year 2024. It is estimated that 400 million yearly infections occur, and currently, half of the world’s population is at the risk of contracting dengue (1, 3). Previously endemic to the tropics and subtropics of Asia, the Americas, the Western Pacific, and Africa; it has now been reported to be rapidly expanding into previously non-endemic regions, such as temperate and even high-altitude regions due to climate change (4, 5). This growing burden of dengue infections highlights the importance of a more comprehensive understanding of the virus, its role in disease progression, and its interaction with the human immune system.

Dengue is an acute febrile illness caused by four types of single-stranded DENVs from the *Flavivirus* genus that elicit distinct serological responses with the antibodies in human blood (6). The four DENV serotypes share about 65% of their genome, with subvariants within each serotype (7). The DENV genome comprises 10 genes coding for 3 structural and 7 non-structural proteins. The three structural proteins are capsid (C), membrane (M), and envelope (E), while the seven non-structural proteins are NS1, NS2A, NSB, NS3, NS4A, NS4B and NS5 (8, 9). The C protein forms the nucleocapsid of the virus, and the M protein plays a crucial role in viral maturation (10, 11). The E protein is essential for viral entry into the host cell by receptor binding and subsequent fusion (11). The non-structural proteins NS1, NS2A, NS4A, and NS4B are involved in the RNA replication process, with NS1 and NS5 also serving as antigens to initiate immune responses in the host cell (9, 12–14). The NS3 protein has helicase and protease functions, while NS4A is involved in autophagy (9, 14).

The replication cycle of the DENV begins with viral binding to the host cell receptors through clathrin-dependent receptor-mediated endocytosis (15). Inside the host cells, the virus undergoes endosomal processing facilitated by Rab5 and Rab7 proteins. Subsequently, the viral genome is released into the cytoplasm and translated into the endoplasmic reticulum (ER). The newly synthesised components are assembled into immature viral particles in the ER undergo maturation in the Trans-Golgi Network via furin-mediated cleavage and are eventually released from the cell through exocytosis (16).

**Figure 1:**
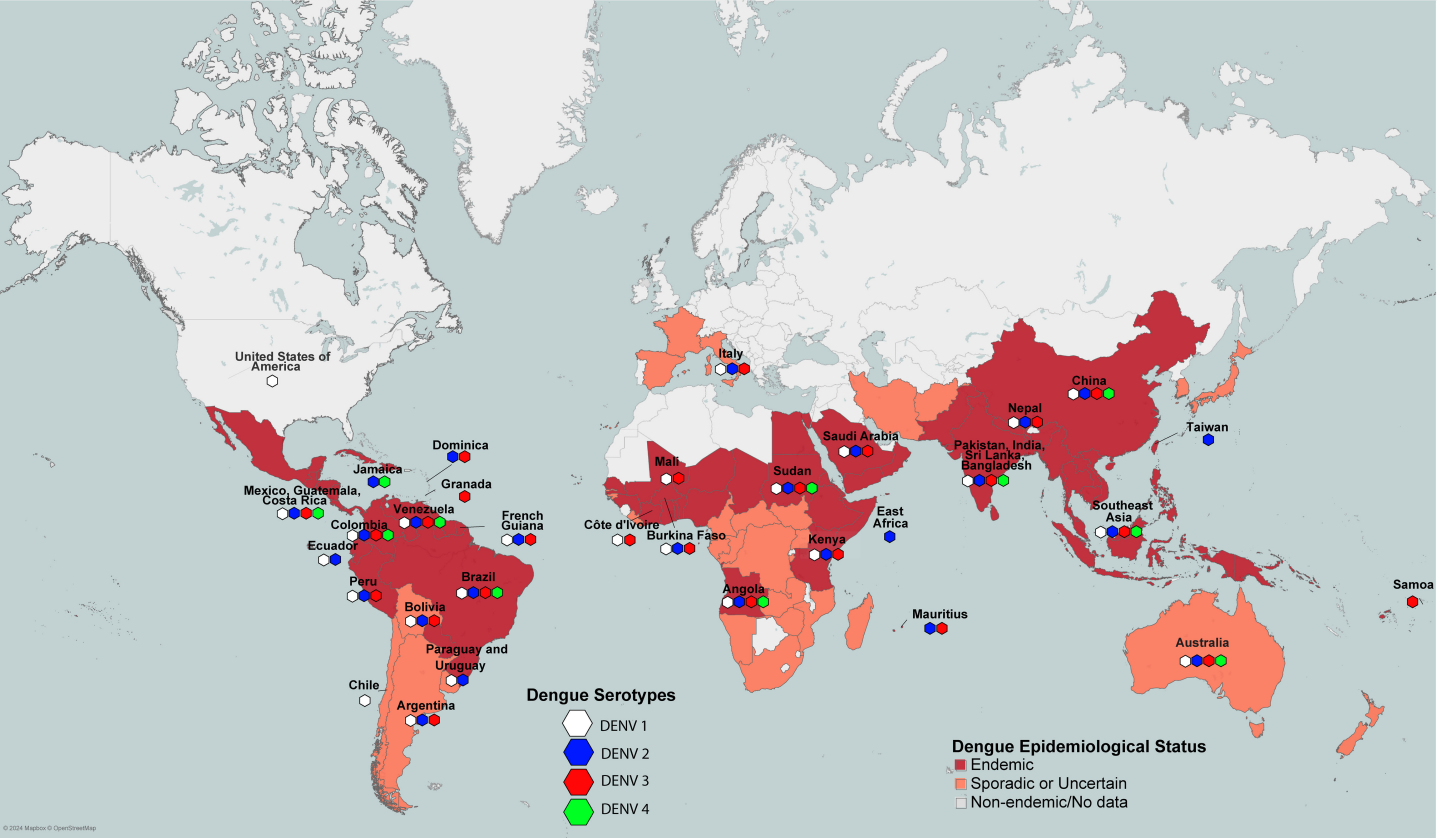
Worldwide pattern of dengue endemicity according to WHO and CDC, and the prevalence of dengue serotypes across different regions.

Four serotypes of DENV have been reported to date (DENV1-4), and each serotype can cause dengue fever (DF), dengue haemorrhagic fever (DHF) (Grades 1-2), and dengue shock syndrome (DSS) (DHF Grades 3 and 4). DENV1-4 are the primary dengue-causing serotypes circulating in humans, with different regions reporting varying profiles of circulating serotypes (**Figure 1**). Common symptoms of DHF include high fever, muscle and joint pain, severe headache, vomiting, bleeding from gums, and skin rashes. Without immediate medical treatment, DHF (Grades 1-2) might develop into DSS, the severe form of DHF, characterized by shortness of breath, rapid pulse rate, low blood pressure and severe abdominal pain. 60-80% of individuals with a primary DENV infection are asymptomatic; however, the risk of severe disease increases significantly during a second infection, especially among those with longer intervals since the primary DENV infection (17). DENV-2 and DENV-4 are more commonly identified in secondary dengue infections, while DENV-1 and DENV-3 often cause primary infections (7, 18).

**Figure 2:**
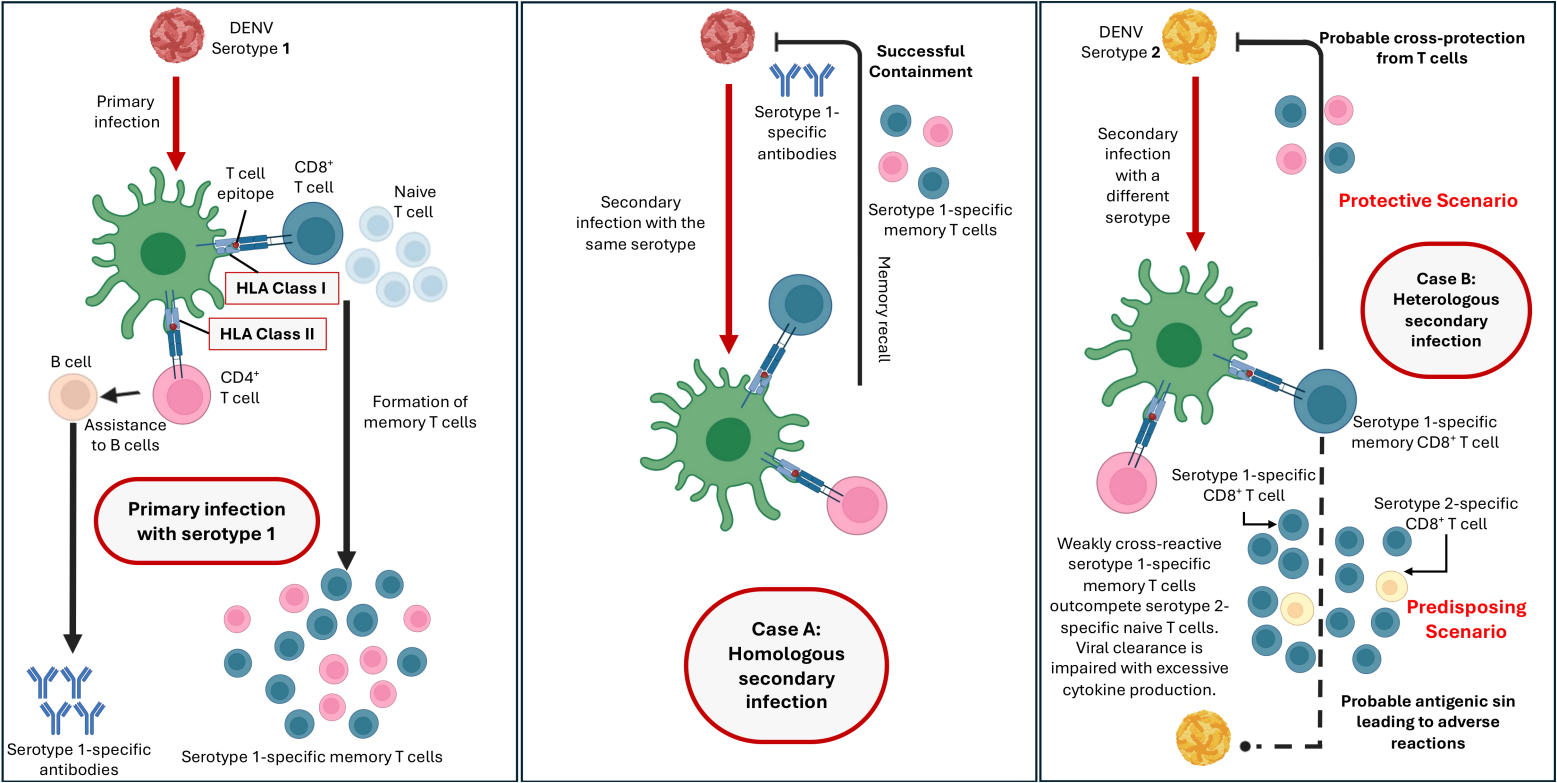
Adaptive immunity mechanisms which could determine the protective and predisposing outcomes against dengue.

There is no consensus on the pathogenesis of DENV infection in the scientific community. The three major factors related to the severity of DENV infection include secondary infection, host genetics, and viral virulence (19–23), with the majority view that secondary infection is the main risk factor for DHF. Primary DENV infection involves the stimulation of interferon gamma (IFNγ) (24). During a secondary infection, non-neutralizing antibodies (25, 26) from the primary infection can bind to the serotype of the second infection. Instead of neutralizing the virus, these antibodies facilitate its entry into host cells, leading to increased viral replication and a more severe immune response. The phenomenon is commonly known as Antibody-Dependent Enhancement (ADE) (27, 28). This heightened immune response can cause increased vascular permeability, plasma leakage, and other severe symptoms characteristic of DHF. The detection of DENV by RIG-I (retinoic acid-inducible gene I) and MDA5 (melanoma differentiation-associated protein 5) triggers the phosphorylation of IRF3 (interferon regulatory factors 3) and IRF7 (interferon regulatory factors 7), leading to the production of type I and III interferons (IFNs), the activation of the JAK-STAT pathways, and the upregulation of interferon-stimulated genes (ISGs) (29). During severe infection, immature DENV particles are recognized by TLR2 (Toll-like Receptor 2) and DC-SIGN (Dendritic Cell-Specific Intercellular adhesion molecule-3-Grabbing Non-integrin) on monocytes and immature dendritic cells, resulting in the release of inflammatory mediators like IL-1β (interleukin 1β) and TNF-α (tumour necrosis factor α), which increase endothelial cell permeability or dysfunction, contributing towards severe complications (30, 31). Moreover, excess secretion of anti-DENV antibodies can exacerbate ADE (**Figure 2**), further increasing autoantibody production and its potential glycosylation, a signature seen in severe DHF and DSS (32, 33).

Endothelial dysfunction could lead to complications such as hypotension, ascites, pleural effusions, shock, and organ dysfunction (34). NS1 protein and inflammatory mediators from monocytes, macrophages, dendritic cells, and mast cells contribute to this dysfunction (34, 35). Elevated cytokines and chemokines, such as IL-1β, IL-6, TNF-α, and MMP-9, directly cause endothelial dysfunction, while others like IL-10 may reduce it (36). Lipid mediators, including PAF, leukotrienes, prostaglandins, and sPLA2 enzymes, also play a role in vascular permeability (36). PAF and prostaglandin metabolites are elevated in severe cases and cause endothelial dysfunction (34, 36, 37). Mast cell products such as histamine, tryptase, chymase, VEGF, and serotonin are linked to disease severity and vascular leakage (38–40). Recent studies from Nicaragua and India showed that both primary and secondary DENV infections can range from subclinical to severe implications (41, 42).

DSS also involves severe cases of hepatomegaly and bleeding, especially in gum, nose, and stool. A typical signature of DSS, hepatomegaly and other complications regarding the liver, high levels of aspartate transaminase (AST) levels are inferred to have played an influential role (43). Secondary hemophagocytic lymphohistiocytosis (HLH), characterized by macrophage activation and cytokine storms, is a significant cause of severe liver dysfunction and high mortality in dengue patients (44, 45). Dengue can cause various bleeding manifestations, including petechiae, gum bleeding, and hematemesis (36). Bleeding results from thrombocytopenia, dysfunctional platelets, coagulation pathway abnormalities, and prolonged shock (36, 46). Over 50% of hospitalized dengue patients experience severe thrombocytopenia, which correlates with disease severity (36). Platelets can be directly infected by DENV, leading to activation through multiple pathways, including serotonin release from mast cells and direct activation by the virus (40, 47).

### 1.2 Human leukocyte antigen and its role in launching immune response against DENV

Since DENV is a foreign body infecting host cells, the genes influencing host defence mechanisms against such foreign infecting agents could play a crucial role in influencing effective response against DENV. One such large complex of genes are the *HLA* genes. *HLA* genes are known to be the most polymorphic regions in the human genome and are the part of Major Histocompatibility Complex (MHC) region located on the short arm of chromosome 6. Class I and class II *HLA* genes encode antigen-recognising sites crucial for adaptive immune responses, recognising between self and non-self. HLA class I proteins present foreign peptides to T-cell receptors on CD8+ T cells (cytotoxic T cells) to launch immune responses. HLA class II proteins are expressed on active immune cells like antigen-presenting cells (APCs) and B cells, where they detect antigens and present them to CD4+ T cells (helper T cells) to initiate immune responses. During Antibody-Dependent Enhancement (ADE) where Fcγ receptors (FcγR) facilitate the increased entry of DENV into immune cells, it could lead to the upregulation of HLA class I molecules, suppressing the activity of NK cells (48). Such reduced activity could potentially contribute to disease pathogenesis. *HLA* class I alleles further influence the magnitude of CD8+ T cell responses against DENV (49–51). Therefore, the host’s *HLA* allele profile is paramount in determining the immunogenic response to DENV infection. This leads to varied immunopathological outcomes, including susceptibility or protection against DENV infection or more severe outcomes (**Figure 2**) (52, 53).

Given the crucial role of *HLA* genes in determining the pathophysiology of DENV infection, numerous case-control studies have investigated how *HLA* polymorphisms influence susceptibility to and protection against severe outcomes of DENV infection. By late 1970s, the idea that HLA proteins could determine the susceptibility towards viral infections started gaining ground (54). *HLA* responses against DENV can also show serotype specificity. A study on the Sri Lankan population has demonstrated that DENV-2-specific responses, likely due to the population’s previous history of DENV-2 infection, signifying that epidemiological history could influence serotype-specific immune responses (51). However, *HLA* allele associations with DENV infection outcomes could be complicated by factors such as *HLA* alleles typing resolution, the population studied, predominant DENV serotypes, *HLA* allele composition, and how the different stages/categories of dengue are defined in the study design. Therefore, usage of standardised pathophysiological classifications for dengue, clear definition of populations studied, and large enough case & control sizes are imperative to derive statistically significant inferences.

The earliest case-control study suggesting the influence of *HLA* alleles in determining susceptibility or protection against DENV infection was performed on Thai children in 1981, identifying several *HLA* class I antigens as potential crucial regulators in the development of severe forms of dengue (55). Throughout the years, several case-control studies, primarily across Southeast Asia, Central and South America, have further cemented the crucial role of different *HLA* class I alleles in determining dengue susceptibility (55–73).

### 1.3 Dengue - an emerging threat in a warming world: understanding DENV and host HLA interactions

**Figure 3:**
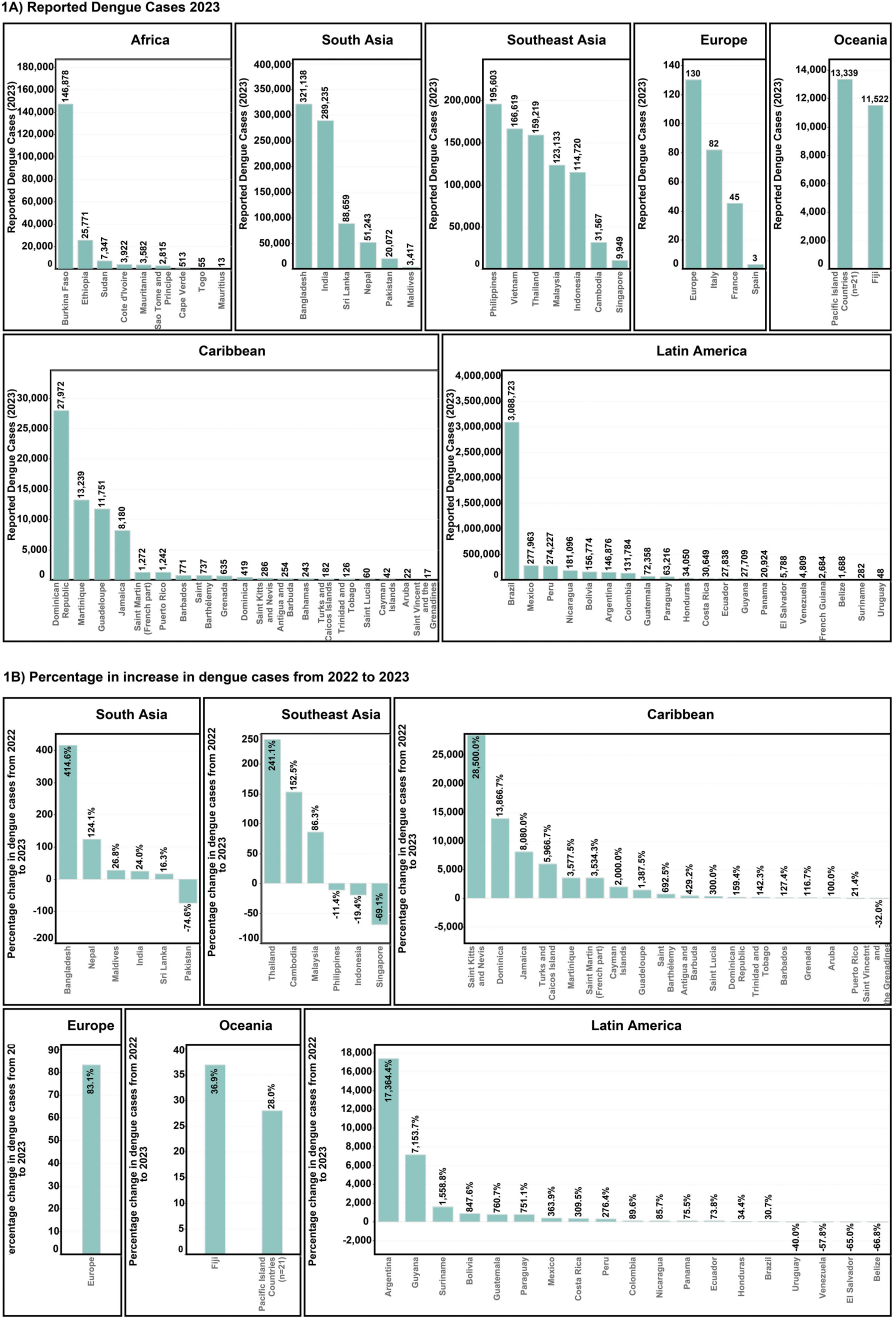
Worldwide dengue case patterns **(A)** Total reported cases for the year 2023 **(B)** Percentage increase in dengue cases from the year 2022-202.

Dengue cases have been rising exponentially over the past five decades with the Americas (Caribbean and Latin American), Southeast Asia, and South Asia, bearing the highest burden of dengue cases (**Figure 3A**). From 2022 to 2023, dengue cases more than doubled in 16 Caribbean and 9 Latin American countries, with St Kitts and Nevis and Argentina reporting increases of 28500% and 17364.4%, respectively. Similarly, cases in Bangladesh in South Asia and Thailand in Southeast Asia showed increases of 414.6% and 241.1% (**Figure 3B**), respectively. In Europe, the locally transmitted cases increased significantly in 2023, with Spain, Italy, and France reporting 3, 82, and 45 cases, respectively (2).

The global increase in dengue cases can be attributed to several factors, including viral evolution (74, 75), human settlement patterns (76), socioeconomic drivers (77), human mobility (78), age (79) and climate change (80). A complex interplay of these factors determines the epidemiological fate of dengue for any given population or geographic setting. Specifically, DENV vectors belonging to *Aedes* sp have rapidly expanded into new habitats, driven by warmer and more humid conditions due to climate change (81–85). Such climatic conditions could result in a shorter extrinsic incubation period (EIP), the period required for a mosquito to become dengue-transmittable after having an infectious meal, increasing dengue risk (86). Furthermore, rising temperatures have been found to increase the biting incidence of dengue vectors, while extended periods of warmer conditions lengthen the suitable period for dengue transmission, heightening the risk of severe epidemics (87).

It is projected that within the next six decades, close to two-thirds of the world’s population will be vulnerable to dengue infection (82). Without adequate preparations, the potential surge of dengue cases could overwhelm medical infrastructures, particularly considering emerging infectious diseases like COVID-19. This growing threat underscores the importance of understanding genetic factors that influence dengue susceptibility and severity.

In the context of the rising dengue cases due to climate change and other anthropogenic factors, we review and perform a meta-analysis of the known associations of *HLA* alleles with dengue. As key regulators of adaptive immunity, HLA molecules present viral peptides to T cells, influencing immune activation and viral clearance. Variability in *HLA* alleles has been linked to differences in dengue susceptibility, severity, and immune response efficiency (88–90). Understanding these associations is crucial to gaining deeper insights into how our adaptive immune system functions and influences DENV infection outcomes.

## 2 Methods

### 2.1 Dengue epidemiological data

Global DENV infection cases and endemicity data were compiled and collected from WHO’s 2024 reports (1, 2) and ECDC (91), latest until 24 June 2024. Missing data from DENV infection cases was collected from the official reported figures mentioned governmental health ministry and infectious disease surveillance website for each respective country (92). We had a total of 75 countries reporting endemic dengue cases until the start of 2024.

### 2.2 Association of *HLA* alleles with dengue worldwide

To look for candidate papers for *HLA*-Dengue association meta-analysis, we searched two databases, PUBMED and Semantic Scholar. In order to make the best use of the two datasets, custom search strategies were used for each, mentioned below:

- PUBMED.
- We entered the following query in the search option to optimise the retrieval of appropriate studies – (Dengue) AND (HLA) AND ((association) OR (associated)). The submitted query returned 101 potential study candidates.
- Semantic Scholar.
- We utilised the elicit (93) tool to streamline the searching process from Semantic Scholar. For a query of “What are the *HLA* alleles associated with Dengue infection outcomes in humans?”, it was ensured that the words “Dengue”, “*HLA*”, “association” or “associated” will should be present within the abstract. This search strategy returned a total of 264 study candidates from an initial 32,604 when searched directly on Semantic Scholar.

**Figure 4:**
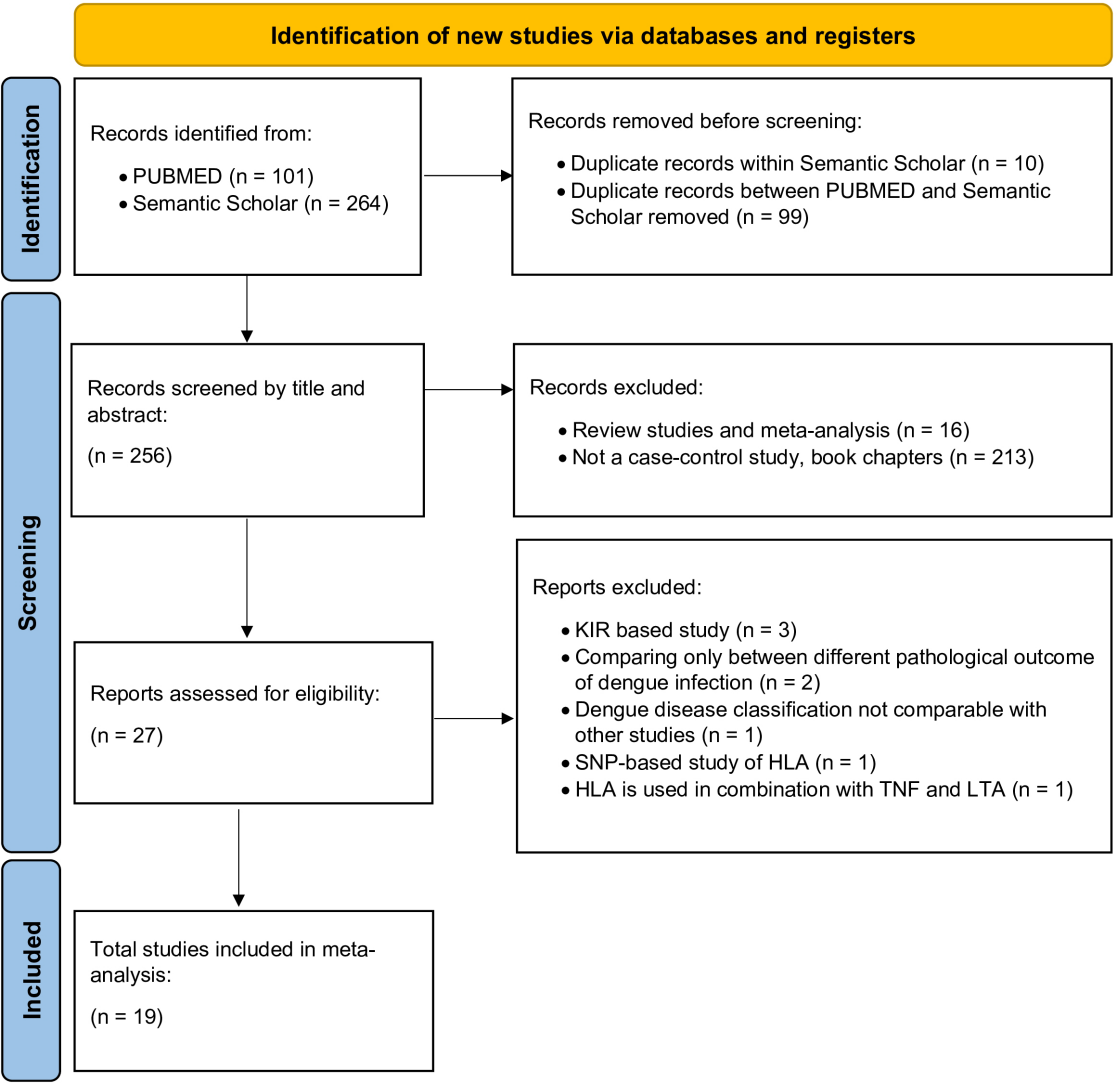
PRISMA flow diagram for the selection of studies for dengue-associated HLA meta-analysis.

With the studies obtained from the above search strategies, we followed the Preferred Reporting Items for Systematic Reviews and Meta-Analyses (PRISMA) guidelines (94) to screen and finalise studies which would be included in the meta-analysis (**Figure 4**). During the selection of the studies to be included for the meta-analyses, the investigators decided on the final set of candidates without any conflicting viewpoints or opinions.

A total of 19 studies have been included in the meta-analysis (**Table 1**), DENV infection phenotypes such as DF, DHF and DSS were comprehensively extracted. Symptomatic DENV infection cases defined as DEN and the sum of DF and DHF was considered as DEN in our meta-analysis. Cochrane RevMan Web (99) was used for calculation of effect sizes of the studies, confidence intervals (CIs), heterogeneity across studies (I^2^ statistics) and generation of forest plots. Fixed-effects model was used to calculate the pooled effect sizes and corresponding 95% CIs. Statistical significance was set at Mantel-Haenszel *P* < 0.05. Forest plots were plotted based on dengue phenotypes (DF, DHF and DEN) and further stratified by populations.

**Table 1:** List of previously reported case-control *HLA* association study with respect to dengue included in the meta-analysis.

No	Country	Study (Author Name, reference)	Year	Population	Cases	Control
*1*	Brazil	Cardozo DM., et al. (59)	2014	Southern Brazilian	95	173
*2*	Brazil	Polizel JR., et al. (68)	2004	White Brazilian	64	667
*3*	Cuba	Sierra B., et al. (69)	2007	Cuban	120	189
*4*	Cuba	Paradoa Pérez ML., et al. (95)	1987	Cuban	82	276
*5*	India	Alagarasu K., et al. (60)	2013	Marathi	114	224
*6*	India	Alagarasu K., et al. (61)	2013	Marathi	114	224
*7*	Jamaica	Brown MG., et al. (72)	2011	Jamaica	50	177
*8*	Malaysia	Appanna R., et al. (67)	2010	Malay, Chinese, Indian	92	95
*9*	Mexico	Falcón-Lezama JA., et al. (70)	2009	Mestizo	39	34
*10*	Mexico	LaFleur C., et al. (63)	2002	Mestizo	81	99
*11*	Philippines	Mercado ES., et al. (56)	2015	Filipino Children	190	300
*12*	Sri Lanka	Weiskopf D., et al. (96)	2016	Sri Lankan	440	150
*13*	Sri Lanka	Malavige GN., et al. (71)	2011	Sri Lankan	110	119
*14*	Thailand	Vejbaesya S., et al. (64)	2015	Thai	440	227
*15*	Thailand	Stephens HA., et al. (97)	2002	Thai	263	140
*16*	Thailand	Chiewsilp P., et al. (55)	1981	Thai	87	138
*17*	Venezuela	Mercedes T. F (98),	2009	Mixed descent Venezuelan	71	127
*18*	Vietnam	N. T. Lan., et al. (62)	2008	Kinh	629	450
*19*	Vietnam	Loke H., et al. (66)	2001	Vietnamese	309	251

## 3 Results

Following the PRISMA guideline (94) (**Figure 4**), a total 19 case-control studies were included in our meta-analysis (**Table 1**). After selecting the candidate studies, we performed a meta-analysis of the most commonly associated alleles across dengue fever (DF), dengue haemorrhagic fever (DHF) or all symptomatic dengue outcomes taken together (DEN). We also conducted a meta-analysis based on the origin of populations to observe susceptibility tendencies at a population-specific scale for each *HLA* allele against DEN.

### 3.1 HLA-A*02

**Figure 5:**
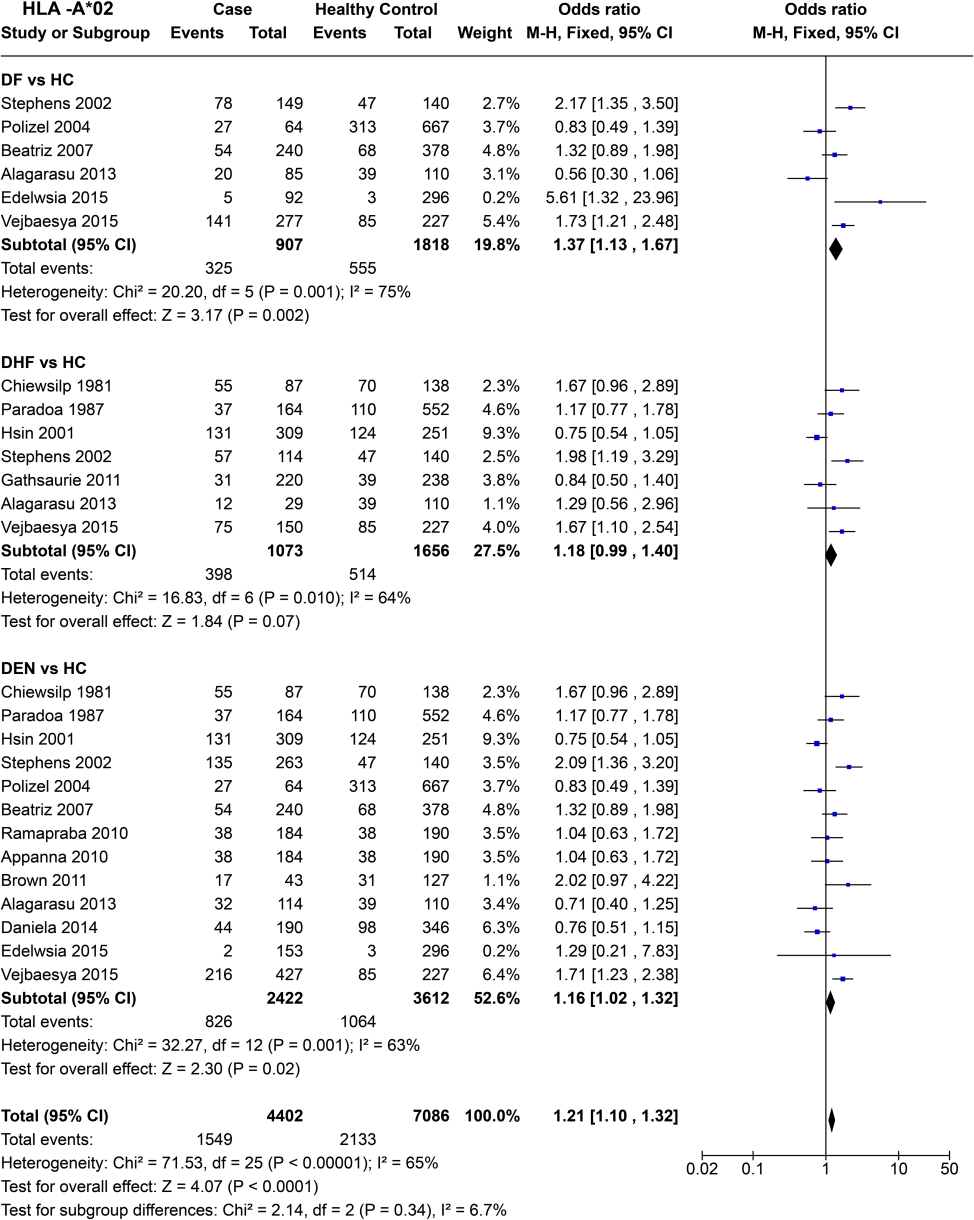
Meta-analysis of HLA-A*02 for dengue fever (DF), dengue haemorrhagic fever (DHF) and DEN (CI, Confidence interval; M-H, Mantel-Haenszel fixed effect test for Odds Ratio).

The HLA-A*02 allele shows a significant amount of heterogeneity (*P* = 1.0E-03) across the five tested studies when DF is compared with healthy controls. Despite such heterogeneity, HLA-A*02 [*P* = 2.0E-03, OR = 1.37 (1.13–1.67)] is inferred to have a predisposing effect towards DF development (**Figure 5**).

When comparing DHF with healthy controls, the data from seven selected studies also show significant heterogeneity [*P* = 1.0E-02]. The combined odds ratio for HLA-A*02 for DHF against healthy controls is insignificant [*P* = 0.07, OR = 1.18 (0.99–1.40)], with a tendency towards being more predisposing to DHF (**Figure 5**).

When comparing DEN with healthy controls based on 13 studies, the combined odds ratio [*P* = 2.0E-02, OR = 1.16 (1.02–1.32)] suggests a predisposing influence of HLA-A*02 despite the significant heterogeneity in the observation [*P* = 1.0E-04] (**Figure 5**).

**Figure 6:**
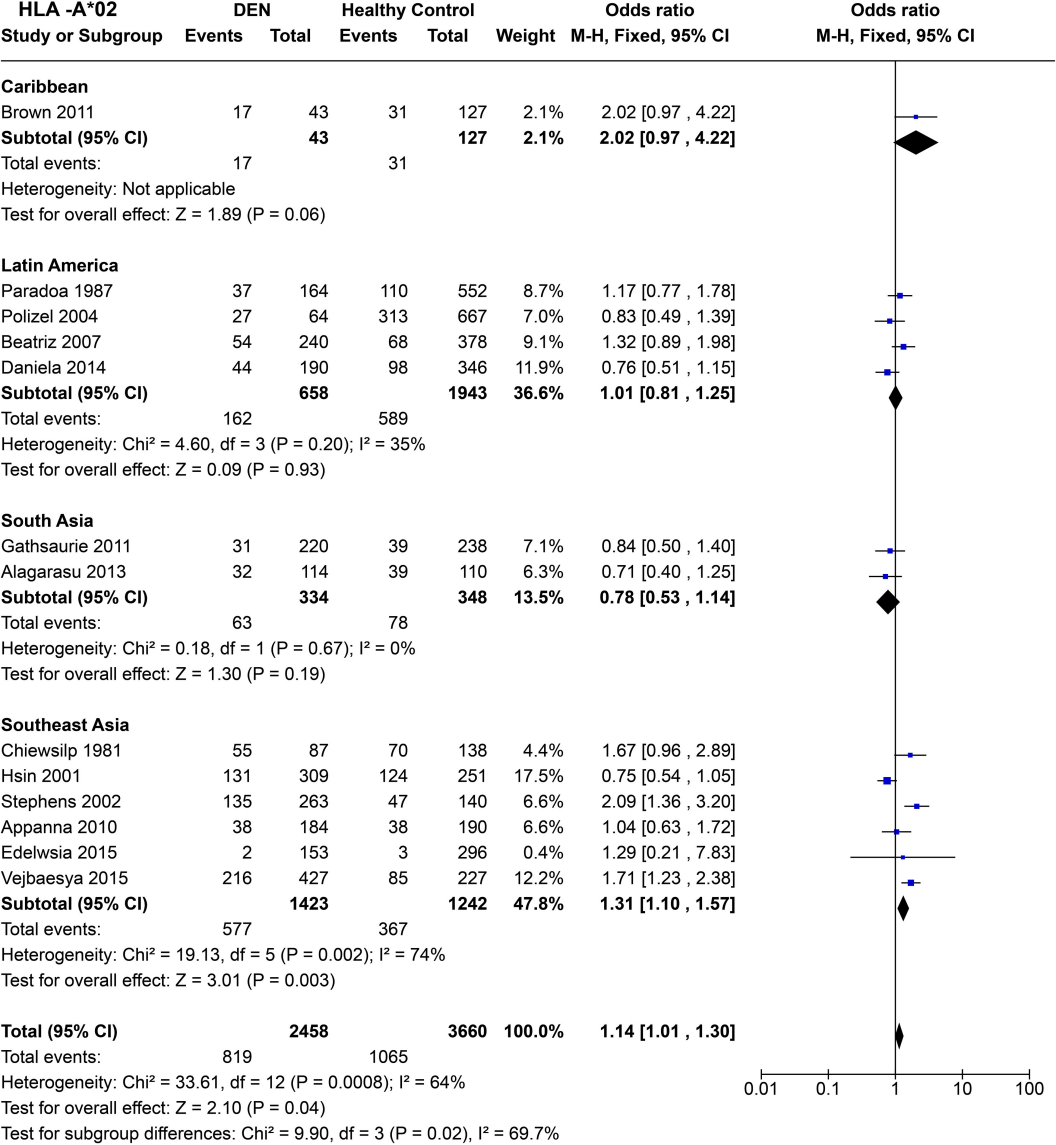
Meta-analysis of HLA-A*02 across different populations for DEN (CI, Confidence interval; M-H, Mantel-Haenszel fixed effect test for Odds Ratio).

To infer the regional tendencies of the HLA-A*02 allele influencing the susceptibility to any forms of DEN, we calculated the odds ratio after grouping the studies based on their region of origin (**Figure 6**). Based on a single study conducted in Jamaica (72), the allele showed a tendency towards being a risk allele with the Caribbean region with the statistics remaining insignificant [*P* = 0.06, OR = 2.02 (0.97–4.22)]. Meanwhile, in Latin America, no significant trend was observed [*P* = 0.93, OR = 1.01 (0.81–1.25)].

In South Asia, the two studies based on Sri Lankan (71) and Indian (61) cohorts reported an insignificant association with dengue with respect to HLA-A*02. The combined odds ratio remained insignificant [*P* = 0.19, OR = 0.78 (0.53–1.14)], with a tendency towards being protective (**Figure 6**). In Southeast Asia, despite significant heterogeneity across studies [*P* = 2.0E-03] driven primarily by the Vietnamese study done in 2001 (66) and the study on Filipino children in 2015 (56), a clear trend of risk emerged. Based on the six selected studies across Thailand, Vietnam, Malaysia, and Philippines, HLA-A*02 is inferred to be a statistically significant risk allele for DEN [*P* = 2.0E-03, OR = 1.31 (1.10–1.57)] (**Figure 6**).

When combined across different regions and accounting for significant heterogeneity (*P* = 8.0E-04), the risk predisposition characteristic of HLA-A*02 stands [*P* = 4.0E-02, OR = 1.14 (1.01–1.30)] (**Figure 6**). It is very likely that this inference is driven primarily by the Southeast Asian studies, given their higher weight in the analysis conducted. Moreover, regardless of significant associations with either being a risk or protective against DEN within each region, the tendency of odds ratio differs significantly between each regional group (*P* = 2.0E-02). This difference among different region groups suggests a population-specific variability of the susceptibility profile of HLA-A*02.

### 3.2 HLA-A*03

**Figure 7:**
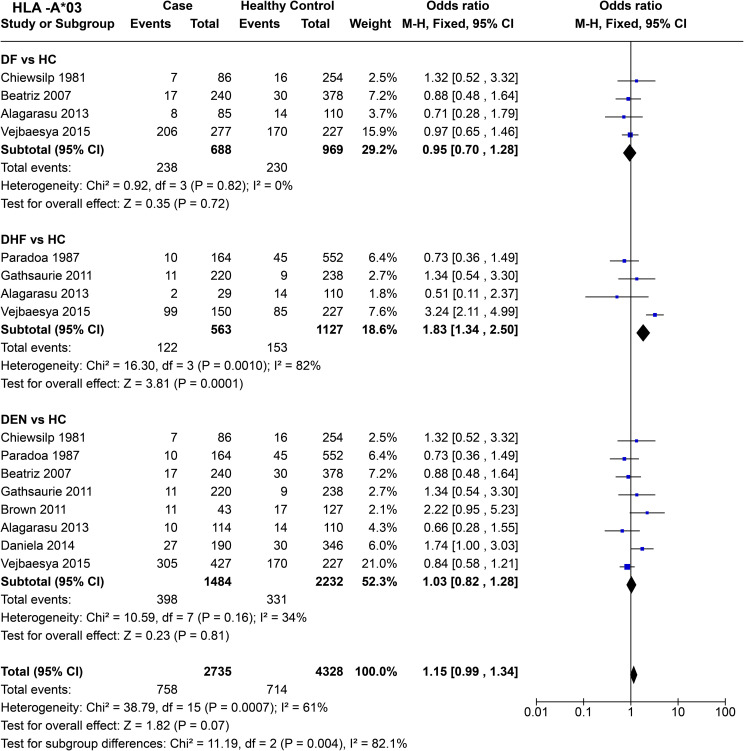
Meta-analysis of HLA-A*03 for dengue fever (DF), dengue haemorrhagic fever (DHF) and DEN (CI, Confidence interval; M-H, Mantel-Haenszel fixed effect test for Odds Ratio).

HLA-A*03 do not show any significant effect on DF susceptibility when compared to healthy controls across four studies [*P* = 0.72, OR = 0.95 (0.70–1.28)] (**Figure 7**). When DHF is compared with healthy samples, significant heterogeneity between the four included studies was observed (*P* = 1.0E-03). Despite this heterogeneity, the combined odds ratio [*P* = 1.0E-04, OR = 1.83 (1.34–2.50)] suggests a predisposing effect of HLA-A*03 towards DHF, primarily driven by the Thai study conducted in 2005 (64).

When DEN was compared with healthy controls, no significant associations towards risk or protection were observed [*P* = 0.81, OR = 1.03 (0.82–1.28)]. Overall, HLA-A*03 shows a statistically insignificant [*P* = 0.07, OR = 1.15 (0.99–1.34)] tendency to be a risk allele. Within the three tested outcomes, DF, DHF, and DEN, a significant difference between each group was observed [*P* = 4.0E-03] (**Figure 7**).

**Figure 8:**
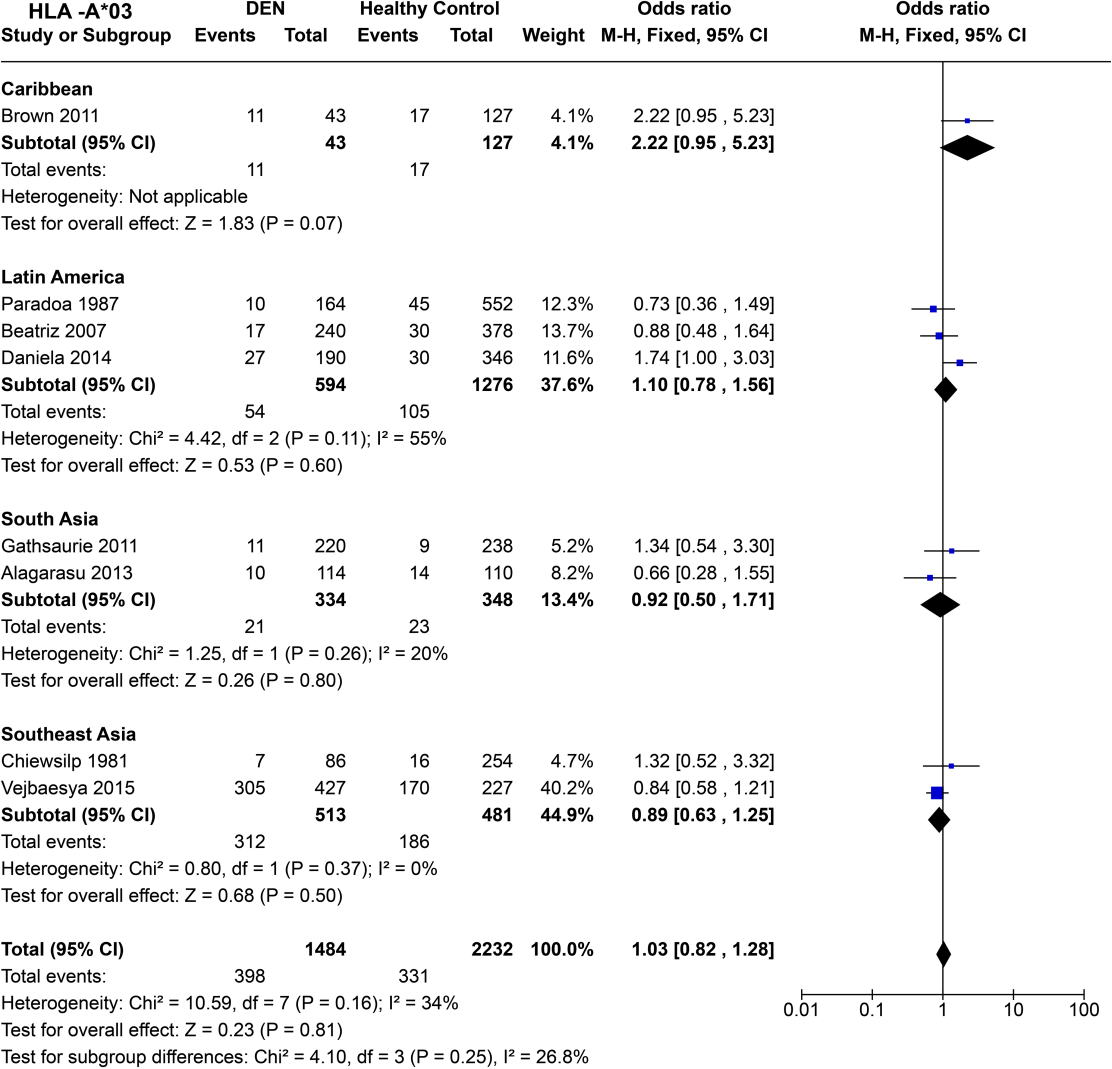
Meta-analysis of HLA-A*03 across different populations for DEN (CI, Confidence interval; M-H, Mantel-Haenszel fixed effect test for Odds Ratio).

Within the Caribbean [*P* = 0.07, OR = 2.22 (0.95–5.23)] and Latin American [*P* = 0.21, OR = 1.12 (0.81–1.56)] populations, statistically insignificant influence of HLA-A*03 on DEN susceptibility was observed (**Figure 8**). In South Asia [*P* = 0.80, OR = 0.92 (0.50–1.71)] and Southeast Asia [*P* = 0.34, OR = 0.84 (0.58–1.21)], no significant influence of HLA-A*03 on DEN was inferred (**Figure 8**). Overall, when all regions are compared, HLA-A*03, does not show any significant effect on DEN susceptibility [*P* = 0.81, OR = 1.03 (0.82–1.28)].

### 3.3 HLA-A*24

**Figure 9:**
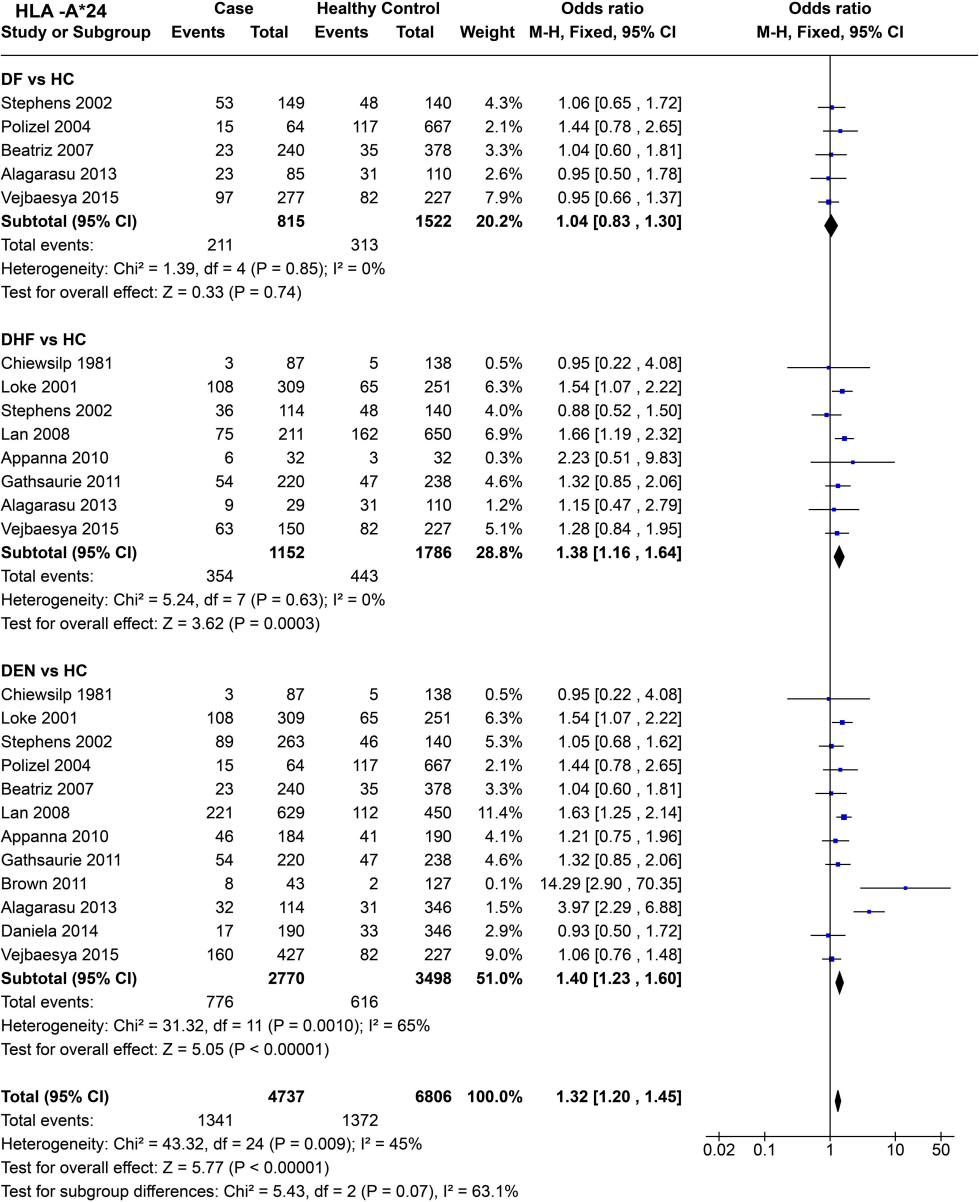
Meta-analysis of HLA-A*24 for dengue fever (DF), dengue haemorrhagic fever (DHF) and DEN (CI = Confidence interval; M-H = Mantel-Haenszel fixed effect test for Odds Ratio).

HLA-A*24 (**Figure 9**) shows no statistically significant protective or predisposing effect for DF [*P* = 0.85, OR = 1.04 (0.83–1.30)]. When we compare DHF with healthy controls across eight studies, the significant combined odds ratio [*P* = 3.0E-04, OR = 1.38 (1.16–1.64)] suggests a predisposing influence of HLA-A*24 towards DHF.

When we compare DEN with the healthy control across 12 studies (**Figure 9**), the predisposing influence of HLA-A*24 [*P* < 1.0E-05, OR = 1.40 (1.23–1.60)] stands despite significant heterogeneity [*P* = 1.0E-03] across different studies.

In terms of combined influence of HLA-A*24 across the three different subtypes of dengue infection, the statistics [*P* < 1.0E-05, OR = 1.32 (1.20–1.45)] (**Figure 9**) suggest an overall predisposing influence towards dengue.

**Figure 10:**
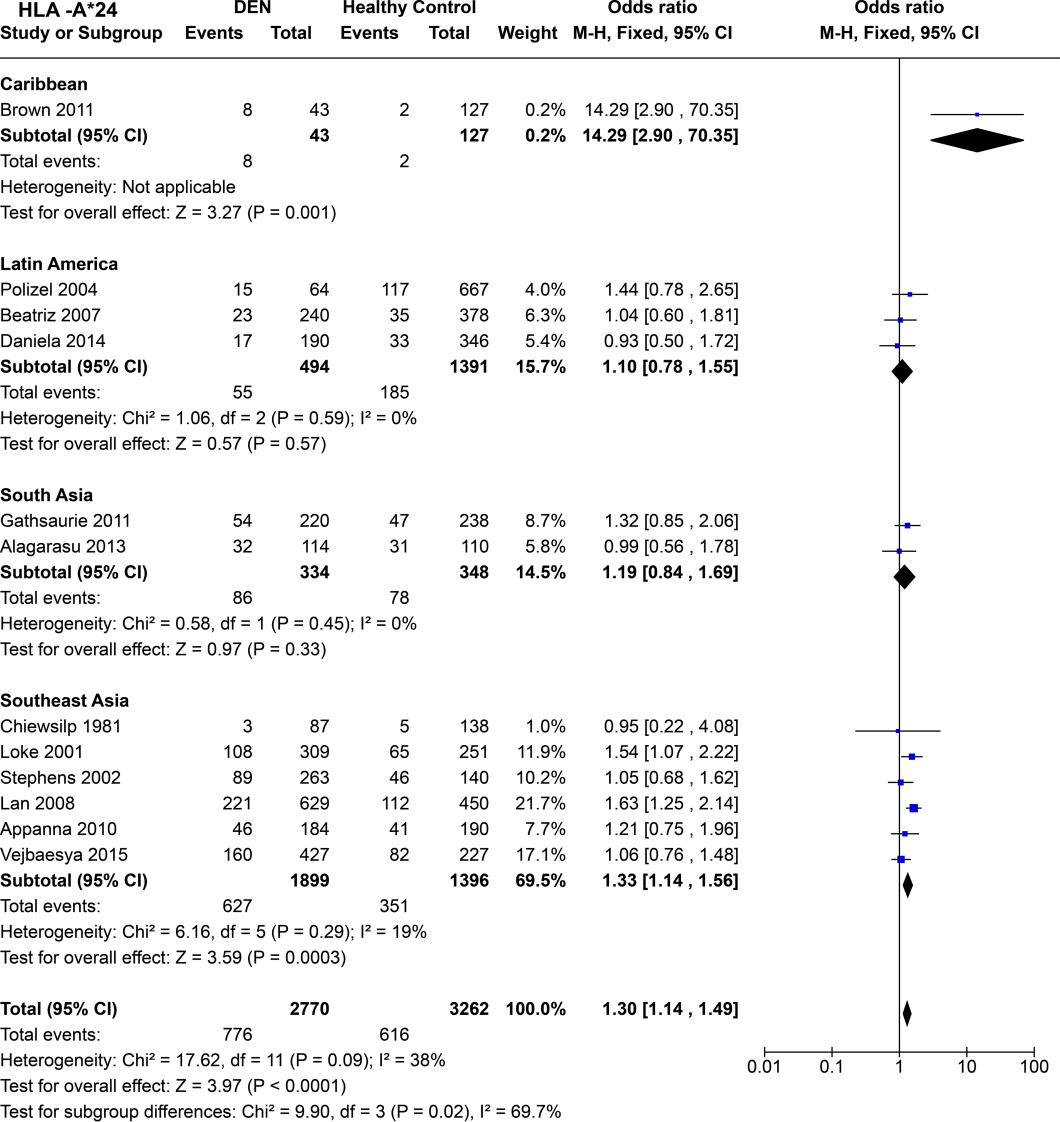
Meta-analysis of HLA-A*24 across different populations for DEN (CI, Confidence interval; M-H, Mantel-Haenszel fixed effect test for Odds Ratio).

In terms of population specific tendencies (**Figure 10**), HLA-A*24 shows significant predisposition towards DEN in the Caribbean population [*P* = 1.0E-03, OR = 14.29 (2.90–70.35)]. It is noted that the wide confidence interval for the study is due to its small sample size and should be interpreted with caution. In the three Latin American studies (59, 68, 69), HLA-A*24 does not show statistically significant associations with being a risk or a protective factor for DEN [*P* = 0.57, OR = 1.10 (0.78–1.55)]. Having said that, the tendency of the allele could be considered towards being predisposed to DEN.

Among South Asians, HLA-A*24 shows a similar trend to that of Latin Americans, with no statistically significant associations towards being a risk or protective factor for DEN [*P* = 0.33, OR = 1.19 (0.84–1.69)], while having a tendency towards being a risk allele for DEN (**Figure 10**). However, in Southeast Asia, HLA-A*24 is inferred to be a risk allele for DEN based on the combined odds ratio [*P* = 3.0E-04, OR = 1.33 (1.14–1.56)] (**Figure 10**).

When all the regional populations are considered together, HLA-A*24 is inferred to be a predisposing allele for DEN [*P* < 1.0E-05, OR = 1.30 (1.14–1.49)] (**Figure 10**). This inference is significantly driven by the outcomes from the studies on Southeast Asian populations, which contributed to a higher number of sample counts.

### 3.4 HLA-A*33

**Figure 11:**
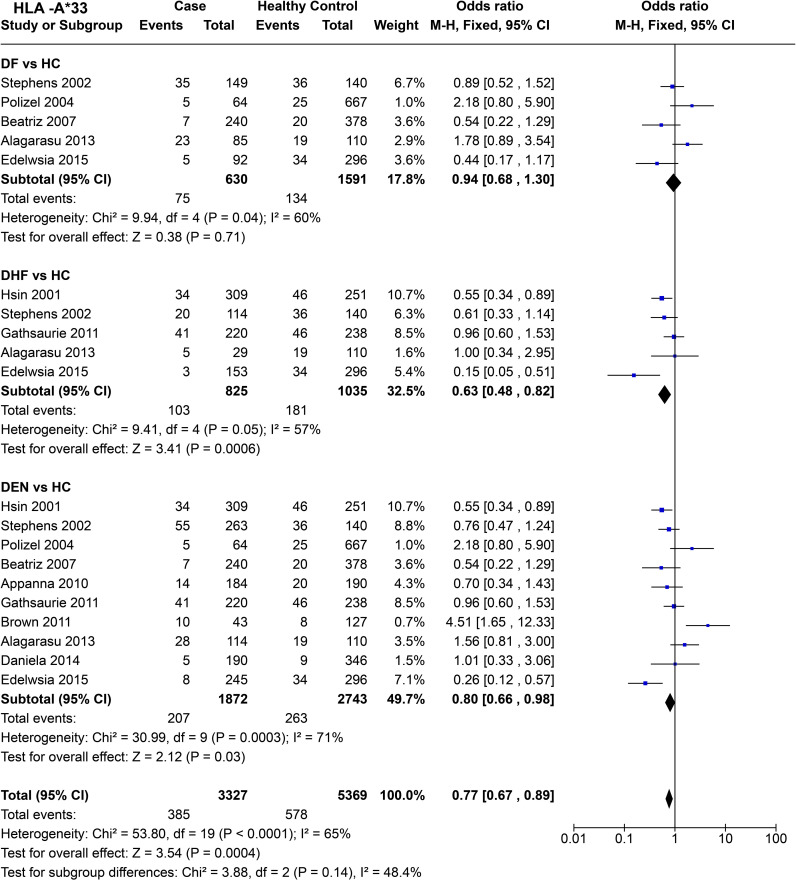
Meta-analysis of HLA-A*33 for dengue fever (DF), dengue haemorrhagic fever (DHF) and DEN (CI, Confidence interval; M-H, Mantel-Haenszel fixed effect test for Odds Ratio).

For HLA-A*33 (**Figure 11**), when comparing DF with healthy controls based on four studies, no significant influence of HLA-A*33 on DF was inferred [*P* = 0.71, OR = 0.94 (0.68–1.30)]. Whereas, when we compared DHF with healthy controls, HLA-A*33 shows significant protective influence against DHF [*P* = 6.0E-04, OR = 0.63 (0.48–0.82)], despite a significant degree of heterogeneity [*P* = 5.0E-02].

Despite significant heterogeneity observed across studies [*P* = 3.0E-04], HLA-A*33 was found to confer a protective effect against DEN [*P* = 3.0E-02, OR = 0.80 (0.66–0.98)] (**Figure 11**). When observed across all the three different tested scenarios for dengue (DF, DHF, DEN), HLA-A*33 was inferred to have a protective effect [P = 4.0E-04, OR = 0.77 (0.67–0.89)] (**Figure 11**). Such an inference could have been driven by HLA-A*33’s stronger protective influence against DHF.

**Figure 12:**
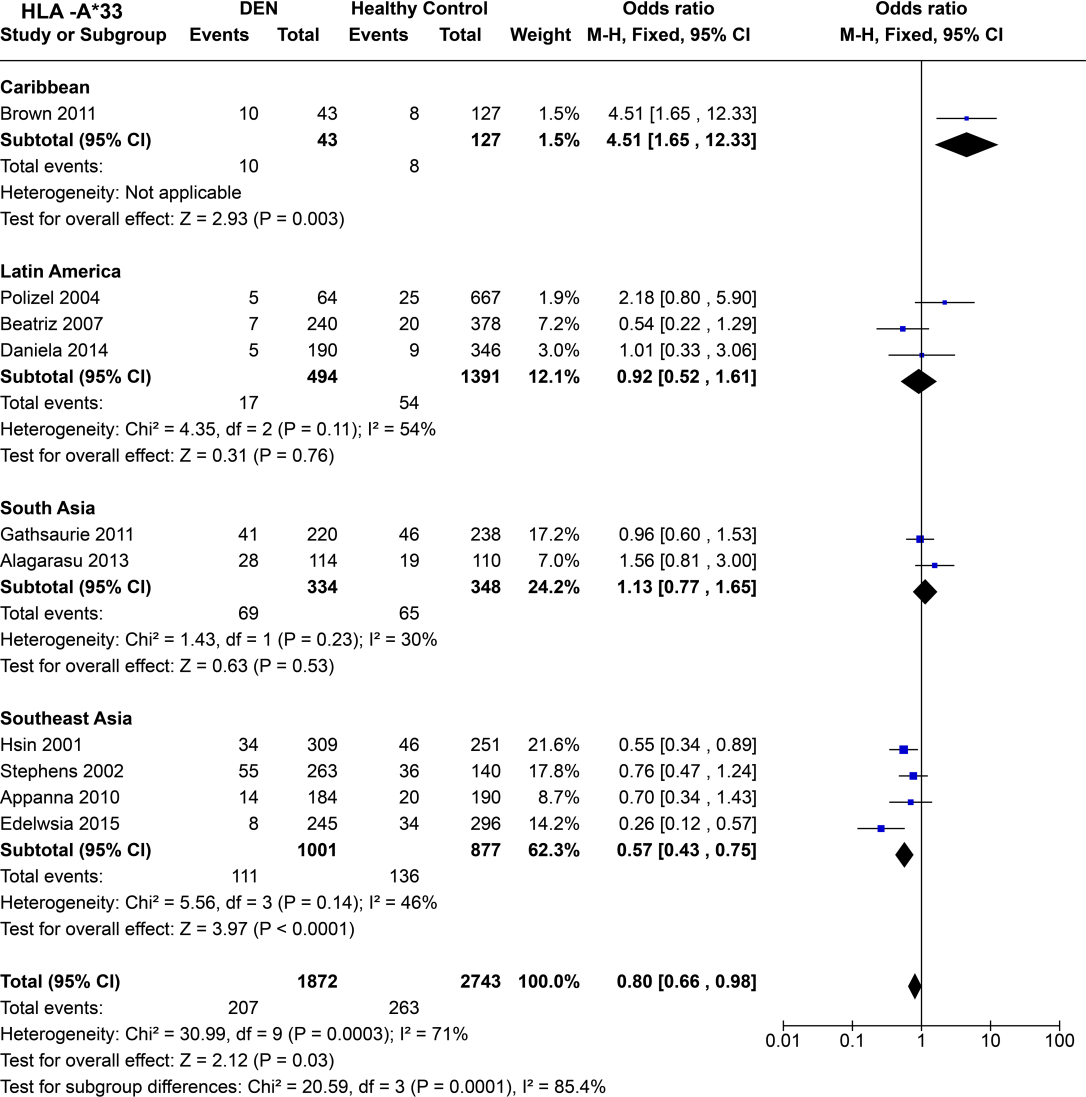
Meta-analysis of HLA-A*33 across different populations for DEN (CI, Confidence interval; M-H, Mantel-Haenszel fixed effect test for Odds Ratio).

When compared across worldwide populations (**Figure 12**), HLA-A*33 is observed to be a risk factor for DEN in the Caribbean population [*P* = 3.0E-03, OR = 4.51 (1.65–12.33)], a trend completely disparate from the general trend observed for HLA-A*33 in the previous set of inference. Furthermore, the small sample size could have played a significant role in the wide confidence interval observed for the Caribbeans. Interestingly, in Latin America, a study on the Brazilian population (68) showed a similar predisposing tendency for HLA-A*33 as the Caribbeans (72), whereas another study on Cubans (69) demonstrated a protective tendency. Overall, HLA-A*33 does not demonstrate any significant protective or predisposing effect with respect to DEN to the Caribbeans [P = 0.76, OR = 0.92 (0.52–1.61)].

Similar to Latin America, HLA-A*33 does not show an association with a protective or predisposing effect towards DEN in South Asians [*P* = 0.53, OR = 1.13 (0.77–1.65)] (**Figure 12**). In contrast, in Southeast Asia, HLA-A*33 is inferred to confer protection against DEN [P < 1.0E-4, OR = 0.57 (0.43–0.75)] (**Figure 12**). When examined individually across the four Southeast Asian studies included in the analysis, the protective trend remains consistent throughout (56, 66, 67, 97).

Considering the four regional population groups together, HLA-A*33 is inferred to be a protective allele against DEN [P = 3.0E-02, OR = 0.80 (0.66–0.98)] (**Figure 12**). This inference is primarily driven by the associations inferred from the analysis of the Southeast Asian populations, having a higher sample size. The statistically significant difference in the combined odds ratio across the different regions further highlights the population-specific difference in the influence of HLA-A*33 on DEN.

### 3.5 HLA-B*44

**Figure 13:**
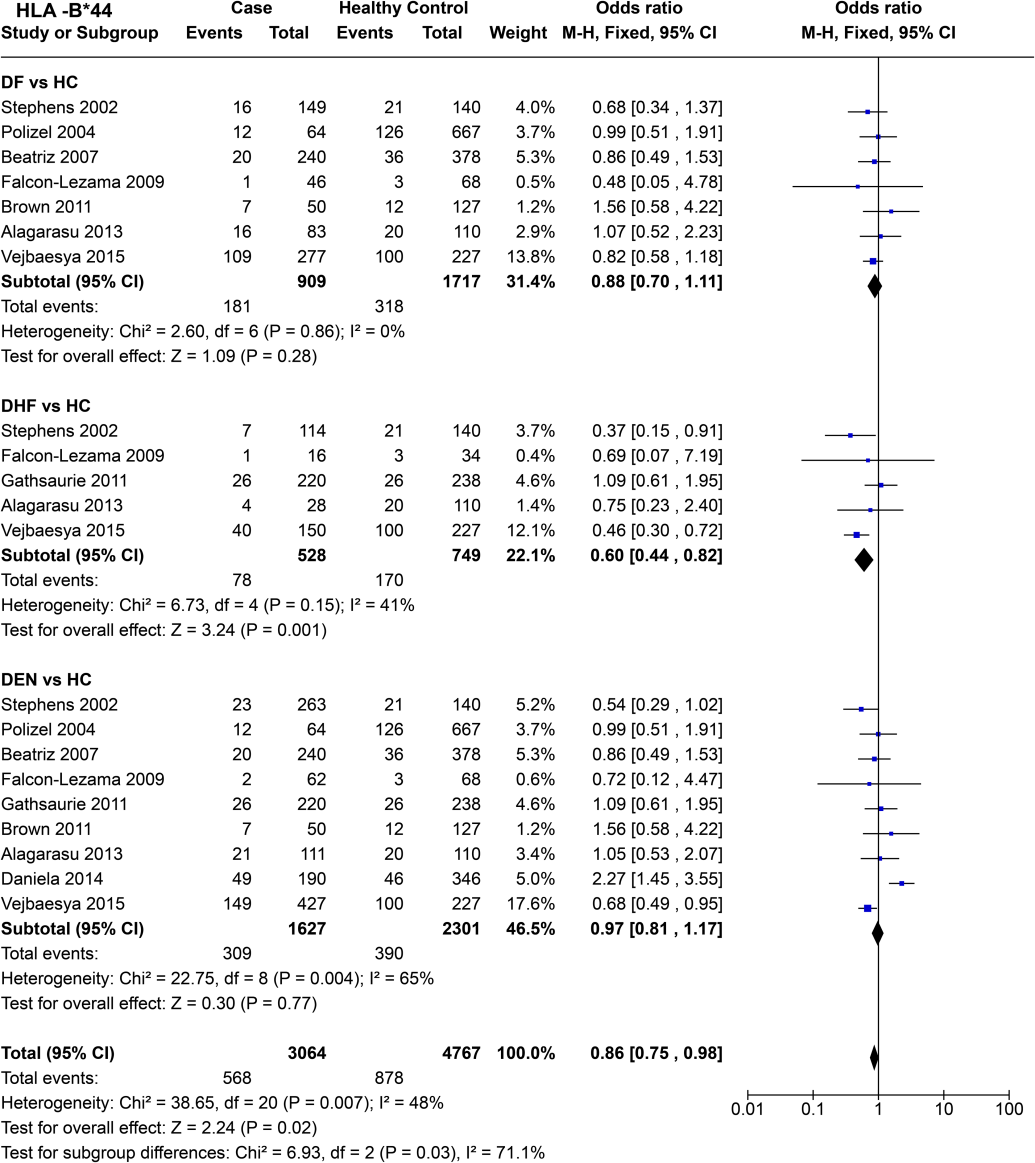
Meta-analysis of HLA-B*44 for dengue fever (DF), dengue haemorrhagic fever (DHF) and DEN (CI, Confidence interval; M-H, Mantel-Haenszel fixed effect test for Odds Ratio).

HLA-B*44 (**Figure 13**) showed no significant protective or predisposing association with DF when compared to healthy controls [*P* = 0.28, OR = 0.88 (0.70–1.11)]. Nonetheless, the observed tendency was towards being protective against DF. When we compare DHF with healthy controls, HLA-B*44 was found to have a protective effect against DHF [*P* = 1.0E-03, OR = 0.60 (0.44–0.82)]. However, this observation does not stand true when comparing DEN with healthy controls [P = 0.77, OR = 0.97 (0.81–1.17)], as no significant protective or predisposing effect is observed.

The overall tendency of HLA-B*44 allele suggests a protective effect against dengue [P = 2.0E-02, OR = 0.86 (0.75–0.98)] (**Figure 13**). The significant difference between each subgroup highlights HLA-B*44’s increased protective influence on DHF likely have influenced the overall role for the allele with respect to dengue.

**Figure 14:**
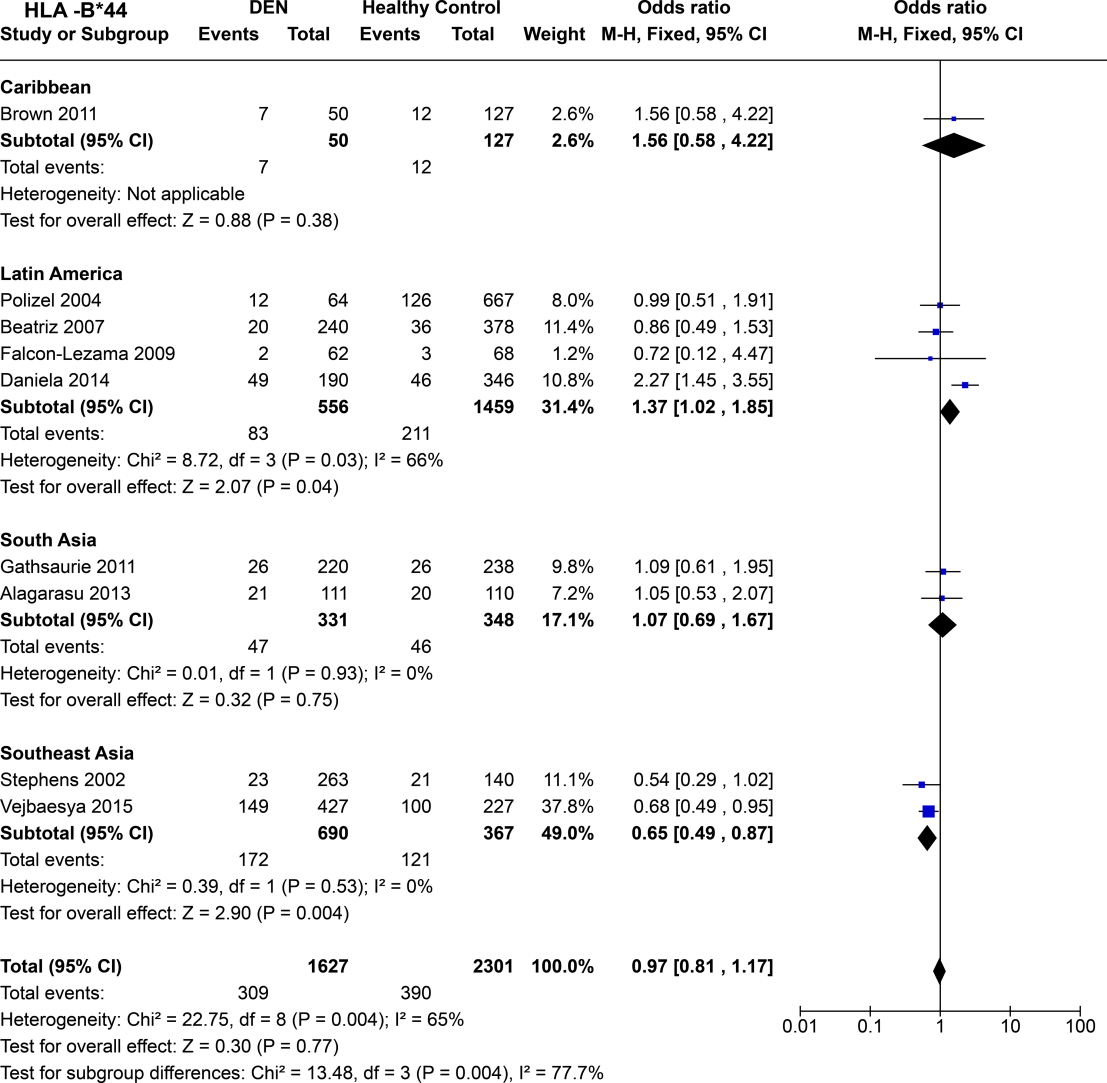
Meta-analysis of HLA-B*44 across different populations for DEN (CI, Confidence interval; M-H, Mantel-Haenszel fixed effect test for Odds Ratio).

Comparing regional population tendencies (**Figure 14**), in the Caribbean, no protective or predisposing influence of HLA-B*44 was observed [P = 0.38, OR = 1.56 (0.58–4.22)], although the observed trend was towards being predisposing. In Latin America, despite showing significant heterogeneity [*P* = 3.0E-02], HLA-B*44 is inferred to have predisposing influence towards DEN [P = 4.0E-02, OR = 1.37 (1.02–1.85)].

In South Asia, HLA-B*44 shows a tendency towards being predisposing; however, no significant association was observed [P = 0.75, OR = 1.07 (0.69–1.67)] (**Figure 14**). In contrast, from the two Thai-based study in Southeast Asia, we can infer a protective effect of HLA-B*44 against DEN [P = 4.0E-03, OR = 0.65 (0.49–0.87] (**Figure 14**).

HLA-B*44 shows no significant association with DEN when all four regions are taken together (**Figure 14**). However, significant heterozygosity [*P* = 4.0E-03] is observed across each region, highlighting the population-specific influence of HLA-B*44 towards DEN.

## 4 Discussion

Given the observed historical trends, dengue could continue its rapid expansion across the globe, potentially increasing the number of people at risk of infection. Countries in Southeast Asia, South Asia, Caribbean, and Latin America share the primary burden of severe epidemics with longer dengue transmission seasons. For instance, Argentina’s severe dengue epidemic of 2023 (**Figures 3A, B**), was characterised by a significantly more humid and hotter summer, leading to more favourable climatic conditions for *Aedes aegypti* to survive (100). Brazil reported 1.5 million cases more in 2023 compared to 2022 (1), driven by fragmentation and degradation of the Amazon for infrastructure development (101, 102) and climate change (103). These factors contribute to the spread of dengue to the most remote regions within Brazil, leaving many previously unexposed populations vulnerable to dengue.

South Asia and Southeast Asia continue to have a significant burden of dengue. Bangladesh recorded its worst dengue epidemic in 2023 with more than 300,000 cases, characterised by 414.4% annual increase (**Figures 3A, B**). Such a severe epidemic was attributed to climate change-related factors in the deltaic and primarily riverine country, leading to the spread of dengue far beyond the urban centres to the remotest rural areas (4). In fact, South Asia and Southeast Asia are one of the most endemic regions in the world, especially India, Sri Lanka, Myanmar, Thailand, and Indonesia (2). A significant and relatively well understood exception in Southeast Asia is Singapore, which reported a 69% drop in dengue cases from 2022 to 2023 (**Figure 3B**) due to effective governmental controls despite increased conduciveness for dengue due to climate change (104).

It has been estimated that dengue cases could rise by 13% for each 1°C increase in temperature (105). Studies have also taken into consideration of GDP and economic growth as predictive variables and suggested future economic growth could counterbalance dengue case increase, while population growth could be the driving factor in case of any increase in the number of cases (106). Nevertheless, climate change could raise dengue risk further in future, having serious implications for human health (3, 81, 106, 107). To mitigate this risk, understanding *HLA*’s role across different populations and dengue outcomes is crucial.

From the meta-analysis, we infer that HLA-A*02 is associated with predisposition towards DF and DEN, while also showing a tendency to be a risk factor for DHF. We demonstrate a significant difference between the tendencies of HLA-A*02 associations towards DEN across different regional populations (**Figure 6**). The predisposition towards dengue infection is primarily driven by the Southeast Asian populations, which signifies that HLA-A*02 could be considered as a risk allele for Southeast Asia in particular (**Figure 6**). Our inferences about HLA-A*02 provide a significant improvement compared to previous meta-analyses by delving into different pathological outcomes and population-specific patterns of associations (89, 90). In Southeast Asia, HLA-A*02 is a common *HLA* allele across numerous populations throughout the region (108), thereby could be considered as a warning signal for preparing effective medical and policy measures to prevent future dengue outbreaks.

We highlight that HLA-A*03 is a risk allele for DHF (**Figure 7**). However, this observation is primarily driven by a Thai population-based study showing a strong predisposition for DHF compared to other studies in the same group (64). However, such inference may not be enough to conclude the mentioned association at the scale of Southeast Asia (**Figure 8**). More studies within Southeast Asia and Thailand are needed to confirm this association.

HLA-A*24 is inferred to be a predisposing allele towards DHF and DEN (**Figure 9**), similar to a previous meta-analysis with smaller statistical power (89). However, we uncover the population-specific association in much higher resolution, indicating that HLA-A*24 could be a risk allele for Southeast Asia, while showing similar tendencies in South America and South Asia (**Figure 10**). In Southeast Asia, HLA-A*24 is relatively common, with the highest frequency within indigenous populations of Malaysia, Myanmar, and Northeastern Thailand, which could make them susceptible to the disease (108). Although showing similar tendencies, the number of South Asian and Latin American studies remain limited, which may not reveal the true tendencies for association.

HLA-B*44 was inferred to have an overall protective association with significant heterogeneity across different studies (**Figure 13**). However, it revealed contrast in association tendencies when the studies were stratified based on regional populations. In Latin America, driven primarily by Daniela et al., 2014 (59), HLA-B*44 showed predisposition towards dengue, with South Asia and Caribbean following the same tendency (**Figure 14**). In contrast, HLA-B*44 is found to be associated with protection against DEN in Southeast Asians, driven by a homogenous tendency within the Southeast Asian studies reporting the allele (**Figure 14**). Within Latin America, this *HLA* allele is most common within mestizos in Cuba, Argentina, Ecuador, and Brazil, especially in the Amazonia region (108). Interestingly, HLA-B*44 is most common primarily in the Iberian Peninsula, which could point specifically to the colonial origin of this risk to those regions.

The general tendencies of *HLA* allele associations could be attributed to the possible results of host-pathogen co-evolution (109). This could be especially true for *HLA*, where there is much support for the idea of pathogen diversity influencing the high polymorphism of the *HLA* gene regions. A previous study inferred lower binding efficiency for *HLA* class I A02 and A24 supertypes for all four dengue serotypes, which could explain their observed predisposing tendency towards DEN (110). Similarly, B44 supertypes demonstrated significantly higher binding efficiency, which could explain its protective association within Southeast Asians, however it fails to explain the contrasting observation within Latin Americans (110). Interestingly, dengue and other flavivirus species showed unique binding characteristics with respect to *HLA* (110).

The overwhelming majority of *HLA* class I alleles influencing dengue risk highlights their crucial role in dengue pathogenesis. It has been postulated that *HLA* class I restricted CD8^+^ T cell lymphocytes determined the immune response and risk towards more DHF. Two ways could mediate such influence by secretion of anti-viral cytokines and cytolysis of infected cells (66). Moreover, secreted inflammatory cytokines can affect vascular cell permeability (111), which could severely damage the cell integrity when DENV epitopes mimic host proteins leading to the loss of self-tolerance of the T cells (112). Such scenarios could lead to serious vascular damage and leaking, leading to DHF complications. Moreover, *HLA* class I alleles were also found to be upregulated when infected with DENV *in vivo*, which led to the suppression of natural killer cell response, suggesting a crucial role in dengue pathogenesis for *HLA* class I alleles (48).

Despite having valuable inferences from previously published case-control studies, we could be missing out on further potentially vital insights on how *HLA* allele variations influence dengue. The main factors contributing to this potential missing out of vital insights is the lack of case-control studies from the majority of dengue endemic regions or usage of small sample sizes in association studies, hampering association powers. Until now, vast region of tropical Africa having dengue endemic regions remains unrepresented. Whereas in South Asia, which has witnessed several dengue epidemics in recent years, there have been limited *HLA* association studies with statistically significant sample size. To our knowledge, studies from a single cohort each from vast countries like India (60, 61) and Sri Lanka (71) are the only studies reporting *HLA* association with dengue. Given the diverse genetic landscapes across such under-represented regions, we may find further crucial insights into how human *HLA* diversity influences dengue risk, which could shape regional medical strategies and deeper understanding of disease pathogenesis. Sufficient sample size for significant association power, proper consideration of demographic profiles of populations which play crucial role in determining dengue risk and pathogenesis, like age (79) and gender (113, 114) (especially considering immune modulation by sex hormones) should be considered and investigated in detail to understand the full spectrum of HLA mediated immune reaction towards dengue. We further suggest applying population genetics approach on a finer scale to determine *HLA*-associated dengue risk characterisation. For instance, in a population with multiple ancestries, will the frequency of dengue-associated HLA alleles vary based on the proportion of any particular ancestral component in the genomic makeup of the samples? This could be more pronounced in the recently admixed populations in the Americas, such as mestizos with Native American, European, and African ancestries. Such inferences may help to shape mitigation strategies based on such risk.

Overall, due to current climate change, the populations at risk in the future are bound to increase by many folds. Without effective preparedness, dengue is bound to have serious negative repercussions on public health, influencing the global and national economy and straining medical infrastructures. Conducting a meta-analysis on *HLA* allele-associated dengue risk, considering the impacts of climate change and evolutionary influences, can yield numerous long-term benefits. Studying the crucial immune gene *HLA*’s associated with different forms of dengue potentially aid in understanding host genetic factors and mechanism influencing the pathogenesis of dengue, leading to improved predictive models and more effective personalised medical approaches. This comprehensive knowledge could guide public health policies, allowing for targeted interventions and informed policymaking, leading to improved regional and national preparedness. For instance, since our study highlighted the population-specific association of *HLA* alleles with dengue, the inferences could be utilised to determine the level of risk within a specific population based on the frequency of such *HLA* allele, aiding in targeted intervention of health policies.

Climate-informed genetic risk assessments, which integrate future climatic projections under various scenarios of climate change, urbanization, and population growth, along with other demographic, environmental, and societal factors, could play a vital role. By considering these elements alongside the genetic makeup of the studied population, these assessments can help understand population-level predispositions to diseases. Such assessments support effective adaptation strategies and resource allocation, bolstering policy advocacy and community resilience. Understanding the relationship between *HLA* binding and the evolutionary conservation of viral proteins can provide insights into host-pathogen interactions, informing the design of more effective therapies and vaccines (4, 7). This guidance could be crucial since no specific treatments for dengue currently exist. Overall, these benefits significantly enhance dengue prevention and control efforts in the context of changing climate and inform medical policies.
